# Human Granulocytic Ehrlichiosis in Estonia

**DOI:** 10.3201/eid0911.030480

**Published:** 2003-11

**Authors:** Tiina Prükk, Kylliki Ainsalu, Ene Laja, Ada Aigro

**Affiliations:** * University of Tartu, Tartu, Estonia; † Internal Medicine Clinic of Tartu University, Tartu, Estonia; ‡ United Laboratories of Tartu University Clinics, Tartu, Estonia

**To the Editor:** We report a case of a 24-year-old woman living in a rural area of Estonia who had weakness, chills, and diarrhea on May 10, 2002. On day 5 of the illness, she was admitted to the Department of Infectious Diseases, University of Tartu, with high fever (38.5°C) and muscle pains throughout her body. Examination showed mild jaundice, painful and enlarged liver, and inability to move. Throat was erythematous, and enlarged lymphnodes were palpatable on the neck.

Laboratory findings included the following: leukopenia 2.04x10^9^/L; erythrocytes 4.08x10^12^/L; hemoglobin 130 g/L; thrombocytopenia 36x10^9^/L; eosinophils 0%; basophils 1.0%; monocytes 7.5%; lymphocytes 38.0%; neutrophils 51.0%; reactive lymphcytes 2.0%; plasma cells 0.5%; C-reactive protein 38 mg/L (normal <5 mg/L); bilirubin 95 μmol/L (normal <17 μmol/L); aspartate aminotransferase 121 U/L (normal <31 U/L); alanine aminotransferase 108 UL (normal <31 U/L); and alcaline phosphatase 200 U/L (normal 35–104 U/L). Ehrlichiosis was suspected by clinical symptoms and leukopenia, thrombocytopenia, and elevated transaminases.

Human granulocytic ehrlichiosis (HGE) is an emerging tick-borne disease described for the first time in 1994 in the United States (*1*). The first European case of HGE was reported in Slovenia in 1996 (*2*). Infection with *Ehrlichia phagocytophila,* the agent of HGE, occurs in areas endemic for *Borrelia burgdorferi* (*3*). In Estonia, Lyme borrreliosis is frequently diagnosed in humans but the occurrence of ehrlichiosis has not been established for this region, despite our having found some seropositive results in Lyme borrreliosis patients (*4*).

This case of ehrlichiosis is the first diagnosed in Estonia. The initial diagnosis was based on a typical clinical spectrum of symptoms and clinical laboratory findings, which are relatively nonspecific, making the diagnosis problematic (*5*). Polymerase chain reaction results for *Ehrlichia* were negative, and we did not find morula in the blood smear. Indirect immunofluorescence assay (IFA, MRL Diagnostics, Cypress, CA) was used as a confirmatory serologic test. However, results of this assay are often negative during the initial phase of the disease (*5*,*6*). On day 7 of illness, the serologic results for immunoglobulin (Ig) M type antibodies to *Ehrlichia* were positive (1:20) and negative for IgG. The diagnosis of ehrlichiosis was established, and therapy with doxycycline was started. After 4 days, the patient became afebrile, and on day 6 she left for home. One month later, the titers of both types of antibodies to *Ehrlichia* were increased: IgM titer was 1:160 and IgG titer was 1:128; 6 months later, IgM antibodies were negative, and the IgG titer remained unchanged.

Our patient had a typical spectrum of clinical and laboratory changes to *Ehrlichia,* but not very specific findings of infection with *E.* *phagocytophila*. The results of IFA, i.e., IgM antibodies in the beginning of the disease and increasing titer of IgG antibodies during the course of the disease, confirmed the diagnosis. Granulocytic ehrlichiosis should be considered in patients with tick-associated fever.
